# Conflicting Aims and Values in the Application of Smart Sensors in Geriatric Rehabilitation: Ethical Analysis

**DOI:** 10.2196/32910

**Published:** 2022-06-23

**Authors:** Christopher Predel, Cristian Timmermann, Frank Ursin, Marcin Orzechowski, Timo Ropinski, Florian Steger

**Affiliations:** 1 Institute of the History, Philosophy and Ethics of Medicine Ulm University Ulm Germany; 2 Visual Computing Group Ulm University Ulm Germany

**Keywords:** personal data, wearable, older adults, autonomy, rehabilitation, smart sensor, machine learning, ethics, access to health care, justice

## Abstract

**Background:**

Smart sensors have been developed as diagnostic tools for rehabilitation to cover an increasing number of geriatric patients. They promise to enable an objective assessment of complex movement patterns.

**Objective:**

This research aimed to identify and analyze the conflicting ethical values associated with smart sensors in geriatric rehabilitation and provide ethical guidance on the best use of smart sensors to all stakeholders, including technology developers, health professionals, patients, and health authorities.

**Methods:**

On the basis of a systematic literature search of the scientific databases PubMed and ScienceDirect, we conducted a qualitative document analysis to identify evidence-based practical implications of ethical relevance. We included 33 articles in the analysis. The practical implications were extracted inductively. Finally, we carried out an ethical analysis based on the 4 principles of biomedical ethics: autonomy, beneficence, nonmaleficence, and justice. The results are reported in categories based on these 4 principles.

**Results:**

We identified 8 conflicting aims for using smart sensors. Gains in autonomy come at the cost of patient privacy. Smart sensors at home increase the independence of patients but may reduce social interactions. Independent measurements performed by patients may result in lower diagnostic accuracy. Although smart sensors could provide cost-effective and high-quality diagnostics for most patients, minorities could end up with suboptimal treatment owing to their underrepresentation in training data and studies. This could lead to algorithmic biases that would not be recognized by medical professionals when treating patients.

**Conclusions:**

The application of smart sensors has the potential to improve the rehabilitation of geriatric patients in several ways. It is important that patients do not have to choose between autonomy and privacy and are well informed about the insights that can be gained from the data. Smart sensors should support and not replace interactions with medical professionals. Patients and medical professionals should be educated about the correct application and the limitations of smart sensors. Smart sensors should include an adequate representation of minorities in their training data and should be covered by health insurance to guarantee fair access.

## Introduction

Regular physical activity reduces the risk of many chronic diseases and can significantly contribute to rehabilitation. Geriatric patients are often affected by reduced exercise capacity, which leads to mobility restrictions and dependence on support in daily life [1]. Diagnostic methods can be used to assess physical activity levels for enhancing rehabilitation. These include patient-reported outcomes and clinical gait analyses. A limitation of the methods currently in use is that the delivered data are often difficult to objectify [2]. To overcome this limitation, technology developers and physicians have begun to use smart sensors [3].

Smart sensors combine the measurement and analysis of data. They can collect a wide range of data and can be used in different application areas [4]. In this analysis, we focus on the ethical evaluation of smart sensors that use inertial sensors and machine learning algorithms to record and analyze complex movement patterns. For this purpose, patients receive wearable inertial sensors that record the acceleration in space. Using machine learning techniques, these inertial data can be assigned to complex movement patterns, such as standing up from a chair, opening a door, or even falling. Thus, it is possible to record the activity patterns of patients and quantify their daily activity [5]. In this manner, clinicians can objectively assess patients’ daily physical activity and identify their treatment needs. Care and rehabilitation measures can be individually adapted, and treatment progress can be documented [6]. Rehabilitation of geriatric patients using smart sensor technology has the potential to increase the quality of life for many patients. However, the recording of such data monitors all daily activities can be negatively associated with patient surveillance.

The high vulnerability of geriatric patients and the special characteristics of machine learning algorithms also raise ethical challenges, which will be discussed in this paper. We concentrate our research on the following question: What are the ethical challenges of using smart sensors and how can they be minimized? Our goal is to identify and analyze the different ethical values associated with smart sensors and their potential conflicts, and based on this ethical analysis, provide guidance to all stakeholders, including technology developers, health professionals, patients, and health authorities.

## Methods

This research is an ethical analysis that aims to examine the ethical challenges associated with smart sensors in geriatric rehabilitation.

### Systematic Literature Search

First, the literature on smart sensors in geriatric rehabilitation was identified through a systematic literature search. We then inductively extracted evidence-based practical implications of ethical relevance through qualitative document analysis. PubMed and ScienceDirect databases were used to identify published literature between January 2000 and November 2020. The search was supplemented by using Google Scholar. The literature search was carried out using the following steps: first, identification and definition of the research question and creation of a search algorithm; second, identification of relevant studies; third, selection of studies; and fourth, reporting of the results in an ethical analysis based on the principle-oriented approach of Beauchamp and Childress [7]. Therefore, we combined 2 research methods that are frequently used to assess the ethical issues of new developments in medical practice: a systematic review of all ethical aspects and a systematic review of all ethical values [8,9].

As smart sensors are a novel technology, common synonyms and related terms have been used to avoid missing relevant literature. The search algorithm combined the keywords *smart sensor*, *wearable electronic devices*, *wearable*, *intelligent assistive technology* and *internet of things* with the keywords *geriatric*, *elderly*, *rehabilitation*, or *dementia* and *ethics*, *privacy*, *empowerment*, *harm*, *caregiver*, *discrimination*, *informed consent* or *autonomy* in the titles and abstracts of articles.

Owing to the limited number of eligible ethical analyses, articles on the use of sensors in the care of older adults, in general, were also included. The results of these articles were translated by analogy to the application of rehabilitation. Articles that discussed only the implementation, development, or technical specifications of sensor technologies or algorithms were excluded. No restrictions on article type were imposed.

**Figure 1:**
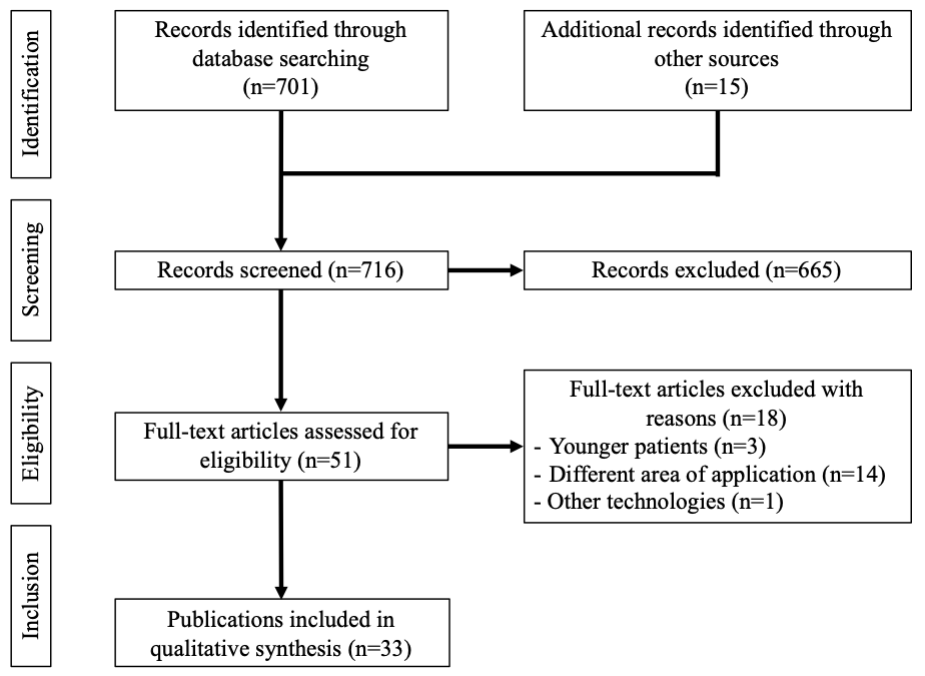
Flowchart of the systematic literature search to identify evidence-based practical implications of applying smart sensors in geriatric rehabilitation resembling the PRISMA (Preferred Reporting Items for Systematic Reviews and Meta-Analyses) statement.

The search algorithm yielded 701 results (Figure 1). Additional 15 articles were identified through hand search using Google Scholar. After removing duplicates and screening the titles and abstracts, 51 articles were considered eligible. After reviewing the full text, 18 articles were excluded because they did not meet the inclusion criteria. The excluded articles focused on younger patients, analyzed different purposes of the application such as sports or lifestyle, or analyzed other technologies, such as robots.

A total of 33 articles were included in this ethical analysis. First, the content of the articles was screened for key information of ethical significance. The content of these articles contains evidence-based practical implications for the application of smart sensors in geriatric rehabilitation.

### Thematic Analysis

Second, a thematic analysis was performed. This is a qualitative approach for identifying, analyzing, and reporting common patterns or themes in narratives or text materials. Articles were explored for recurring themes with a focus on the different values and aims of smart sensors in geriatric rehabilitation [10,11].

### Ethical Analysis

Third, the identified practical implications of ethical relevance were grouped, and an ethical analysis was conducted using the principle-oriented approach of Beauchamp and Childress [7]. If an ethical issue could be examined under more than one ethical principle, we opted to report the issue under the principle that was better suited to highlight ethical conflicts. For reducing biases and omissions, the included articles were critically examined by at least two authors, as recommended for systematic reviews of normative literature [12,13]. We pooled the main ethical issues together after an exchange between the authors in dichotomous pairs of conflicting aims and values. In the following sections, we propose an assessment tool for the ethical evaluation of smart sensors in geriatric rehabilitation. With our tool, physicians, along with their patients, will be able to assess which values are more important to them in each individual case and then weigh the different values against each other.

## Results

In this section, we report the ethical challenges identified in the systematic literature search, grouping them under the 4 principles of biomedical ethics: autonomy, beneficence, nonmaleficence, and justice.

### Autonomy

Respect for autonomy is a fundamental principle of biomedical ethics and requires ensuring that the patient’s will is respected, unless it is in direct conflict with other fundamental values and professional duties. It includes the negative obligation to not constrain a patient’s actions unnecessarily and the positive obligation to disclose information that fosters decision-making. Measures that empower patients tend to increase their autonomy, whereas interventions that directly restrict their liberties or make them hesitant to act freely, restrict patients’ autonomy.

The use of smart sensors in rehabilitation can empower patients by increasing their proactive participation in diagnostics and allowing them an independent life at home. Patients who were asked about the use of wearables in rehabilitation indicated that they expected to be empowered by this technology to manage their own health conditions more effectively [14]. Continuous feedback on the progress of the rehabilitation can motivate patients to become physically active and continue therapy [14-16]. Furthermore, it could provide patients with a deeper understanding of their illness and physical condition [17]. Efficient rehabilitation, aided by smart sensors, can reduce the need for long-term care. Rehabilitation can be supplemented by fall detection and home monitoring, enabling patients to stay at home independently for longer [18,19]. In addition, 58% of patients using fall detectors had improved independence and 72% felt more confident [20]. Through their proactive participation in health management and the possibility of living at home independently for a longer time, patients’ autonomy is increased by the use of smart sensors.

### Privacy

Privacy can be defined as an interest, or even as a right, to be free from intrusion in personal matters, unless major public interests justify such an invasion [21]. When using smart sensors, the protection of privacy requires a person to be left alone when asked and not be monitored without expressed wishes. In contrast, data privacy is concerned with the sensitive handling of data, including their access and use by third parties [22]. Privacy concerns are one of the biggest hurdles for patients in the application of supportive technologies [23]. In a study, Canadian stakeholders were interviewed regarding the challenges of active assisted living technologies. In 30% of the mentions, privacy and security were identified as primary issues [24]. Monitoring patients’ day-to-day activities is highly intrusive. The feeling of being constantly monitored and ubiquitous medical diagnostics can lead to stress and anxiety and may compel patients to adapt their behavior. The evaluation of all daily activities and a desire to achieve good measurement results can lead to excessive physical activity. In a study in which the daily physical activity of patients with chronic obstructive pulmonary disease was measured using sensors, it was shown that participants had a 26% higher activity than the average during the first few days monitored with sensor technology [25]. In the context of rehabilitation of geriatric patients, this may lead to stress and overload symptoms. In consequence, injuries and falls can occur more often.

Patients’ perceptions of privacy loss are significantly influenced by the intrusiveness of the technology used [18,26]. Owing to their low-threshold use, smart sensors offer the potential to minimize the feeling of surveillance through a low degree of intrusiveness and by only collecting data related to preselected complex movement patterns. Studies have shown that patients using smart sensors do not feel violated in their privacy [27]. Patients prefer sensors that can only monitor whether they are active and do not identify specific activities [28]. In most cases, it is not clear whether, to what extent and by whom, the gathered data could be analyzed to conclude information about patients that was not willingly shared by them.

Patients must be informed of the conclusions drawn from the data. From movement data, it is possible, for example, to analyze how often patients use the bathroom, whether they drink alcohol, or whether they are sexually active. It must be discussed with the patient which activities should and could be tracked. Patients should be trained to switch off or dismount sensors when privacy is desired so that they are free to undertake the activities they value and do not have to make unnecessary sacrifices to maintain an image of themselves that they are comfortable sharing with the medical team. As nonmaleficence demands not depriving people from a good they value, loss of privacy can also be seen as a form of harm [29].

### Shared Decision-making

When adequately introduced, the use of smart sensors can improve the *patient-medical professional* relationship and increase autonomy by strengthening the patient’s role as an equal partner. Medical professionals and patients can make therapeutic decisions together, based on data collected by the patient [16,30,31].

Empirical studies have assessed the impact of smart sensors on patient-physician relationships. Patients were asked whether they expected a change in their relationship with their medical professional through wearable technology during rehabilitation. They stated that they expected an improvement in communication and a more patient-centered consultation due to the improved and objective data gathered on their activities [14]. Patients using smart sensors expressed that they were well informed and that decision-making between medical professionals and patients could be improved [32]. Furthermore, the diagnostic process is no longer limited to a visit to the medical practice or hospital; it also takes place beyond these settings. Thus, patients can receive medical support in everyday life [33]. Smart sensor technology used at home can be designed to facilitate contact with medical professionals [34,35].

### Beneficence

#### Overview and General Aspects

The principle of beneficence dictates the orientation of health professionals’ actions toward the well-being of patients. This demands that health professionals make use of both their professional and interpersonal skills to improve the situation of patients, particularly in helping them to fulfill their wish to live in their own homes, while ensuring that such choices do not come at the cost of losing all types of bonds with them.

An advantage of smart sensors is the possibility of independent home monitoring. Long-term home monitoring can provide objective movement data on patients’ everyday life. This can increase the well-being of patients by allowing them to remain in the comfort of their own homes, but it can also reduce the number of social contacts [27,36]. In addition, sensors can contribute to patient safety by extending the monitoring phase after surgery or by identifying patients at a risk of adverse events.

#### Objective Assessment of Daily Activities

Previously, therapy requirements and progress have been determined using gait analyses or patient-reported outcomes. These have high inter- and intraobserver variability and are mostly carried out in a clinical setting [37]. In contrast, sensor technology offers the possibility of objective long-term home monitoring. This has the advantage that the complex movement patterns of patients can be analyzed in everyday situations and over a longer period [38]. Decisions for or against a rehabilitative measure can therefore be made from a broad database. Patients can receive therapy adapted to their everyday life [16]. Thus, autonomy and independence can be promoted in everyday life to benefit patients.

#### Extended Monitoring and Injury Prevention

The use of smart sensors can lead to more individualized therapies for geriatric patients in their homes. Geriatric patients have a high acceptance of sensory technology for long-term home monitoring during rehabilitation [28]. After surgery, patients often remain in the ward for several days for monitoring. Sensor technology can significantly extend monitoring time without the need to keep patients hospitalized. Thus, treatment needs, which only become clear in the patient’s everyday life after discharge, can be identified. Patients benefit from greater security without having to spend more time in the hospital. Owing to the low-threshold use of smart sensors, opportunities for screening and prevention have expanded. People who are expected to need treatment in the future because of hospital stays, comorbidities, or old age can wear sensors in their everyday life. If conspicuous movement patterns appear, practitioners can be informed, enabling them to assess an intervention or rehabilitation need. Thus, the user can benefit from preventive intervention [3]. Furthermore, sensor technology can be used by risk groups to identify and prevent critical events, such as falls [39]. For many patients, an increased sense of security is one of the main reasons for using sensor technology [40,41]. A total of 85% of patients who used a fall detector stated that it improved their safety [20].

The large amount of data collected by smart sensors can be used by machine learning algorithms to detect different anomalies and then take early steps to address health threats. If a patient goes to the bathroom more often than usual, it could be a sign of urinary tract infection or diabetes mellitus. A decrease in the number of outdoor activities could be a result of depression. It must be determined which activities the sensor technology should record and whether findings must be interpreted as relevant for rehabilitation.

### Nonmaleficence

The principle of nonmaleficence indicates that new medical technologies should not disadvantage or harm patients through medical intervention or even diagnosis. The biggest threats to using smart sensors in geriatric rehabilitation are the misuse of patients’ private data and the uncritical acceptance of data provided by the sensors. A major threat to patients is the misuse of data by unauthorized persons. Cyberattacks can steal data from various devices and servers. Owing to the interconnectivity between smart sensors and digital health records, as well as the multiple users and use outside of protected hospital networks, smart sensors represent vulnerable targets for cyberattacks [42].

### Accuracy

In the detection of complex movement patterns, inaccurate activity detections can occur and cause harm to patients. Algorithms may not recognize or they may misclassify movements [19]. Incorrectly classified events can lead to an overestimation of patient’s health. Conversely, the need for rehabilitation or lack of therapeutic success can be overlooked [17]. Therefore, uncritical acceptance of movement data by medical professionals poses a risk to patients. Sensors can support the medical professional’s subjective assessment of care needs with objective data, but cannot replace a complete examination [43]. By increasing the autonomy of patients, there has also been a shift in the roles of patients and medical professionals. The patient is the one who has to apply the sensor technology. As a result, the expectation is placed on the patient to provide high-quality data. Therefore, patients gain more responsibility in the diagnostic process. This could lead to more autonomy but could also jeopardize data quality [44].

### Missing Holistic Assessment

By reducing direct contact with medical professionals and relying more on smart sensors, there is a risk of patients being reduced to the data collected [17]. Social contact with medical professionals is an essential component of therapy. Collecting data on only one physiological parameter, such as movement patterns, does not provide a holistic assessment of health conditions and the rehabilitation process. A holistic assessment can only be discerned through direct interaction with health care professionals [27,34]. Successful treatment requires contact with a medical professional who communicates the results of a diagnosis with empathy and is aware of the patient’s circumstances [30]. The feeling of being monitored can reduce the trust between patients and medical professionals and the acceptance of sensor technology. Moreover, patients may overestimate the accuracy and potential of smart sensors [19].

### Justice

#### Overview and General Aspects

The principle of justice refers to 2 distinct principles: first, that like cases be treated alike and second, to a fair, equitable, and appropriate distribution of health care in society. This demands that every patient should have adequate access to essential health care, regardless of gender, ethnicity, sexual orientation, religion, age, or socioeconomic status [7]. Smart sensors are expensive and can therefore lead to discrimination on the basis of socioeconomic differences. Owing to the dependence on the accuracy of the training data, algorithmic analyses could lead to a discrimination against minorities that are underrepresented in the training data. Geriatric patients who have less experience with technical tools can be at a disadvantage. Conversely, patients living in underserved regions may benefit from the use of sensors in combination with telemedicine. In addition, the success of rehabilitation measures aided by smart sensors depends on the capability of users to use digital technologies, or more broadly, their digital literacy.

#### Socioeconomic Differences

High prices during early technology adoption lead to inequalities in access to personalized rehabilitation. For many patients, the acceptance and adoption of sensors depends on their cost [45]. The use of wearable sensors such as smartwatches shows major demographic and socioeconomic differences. Mainly young, wealthy people buy smartwatches [23]. An additional negative consequence is that smart sensors are optimized on these early adopters, basing the algorithms and the design of the software and hardware on a subpopulation that does not reflect the diversity of the population with rehabilitation needs. New developments that do not solely rely on external systems and are adapted for the geriatric population could overcome this limitation. If sensors are not covered by health insurance and must be purchased by the patients themselves, there will be major inequalities in the medical care of the population [46].

#### Discrimination of Vulnerable Groups

The diagnostic accuracy of smart sensors and the algorithms used by them depends on the training data. Thus, there are differences in accuracy depending on the population group. Population groups that are underrepresented in the training data do not benefit from a high algorithmic output accuracy. They must adapt to the standard defined by the training data even if their movement patterns are normal for their group [17,47]. Furthermore, the movement patterns of men and women differ in some aspects. An algorithm trained using male movement data has a higher output accuracy for men than for women. Similar conclusions can be drawn for other population groups, such as older adults. Studies have shown that it is possible to predict the gender and age of participants with inertial data from gait analysis [26].

There is also a risk of disparity between age groups. The use and function of technical devices are difficult to understand for many older adults. The application of smart sensors in the context of geriatric rehabilitation requires extensive training and education of patients so that they can learn the limitations and correct application of sensor technology and thus benefit from its advantages [46]. Monitoring technologies can cause feelings of stigma and frailty in geriatric patients [27]. Their use can be seen by patients as an admission of frailty and illness to themselves and the social environment [48]. Wearing sensors in public can reveal illnesses or disabilities to strangers [27,49]. In order to mitigate this, smart sensors can be integrated into clothes or smart watches [50]. By giving patients the opportunity to choose between different types of application, the feeling of stigma can be actively reduced.

#### Increasing Numbers of Patients Can Be Treated

Smart sensors have the potential to provide high-quality care to each patient. The quality of human-influenced treatment depends heavily on the experiences, prejudices, and daily constitution of medical professionals. Smart sensors developed and evaluated in congruence with ethical principles offer the possibility of consistently delivering high-quality treatment [51]. Owing to automated data collection and processing, smart sensors offer the possibility of treating more patients at a consistent and even higher quality of care. In many places, there is a supply gap between urban and rural areas in specialized medical care. By using smart sensors in combination with telemedicine, patients in underserved regions can be connected to medical specialists [3]. As previously discussed, this requires extensive training, which not all patients, especially geriatric patients, can follow. Furthermore, fair access to new promising technologies, such as smart sensors, must be guaranteed in rural areas.

## Discussion

### Principal Findings: Conflicting Aims and Values

**Figure 2:**
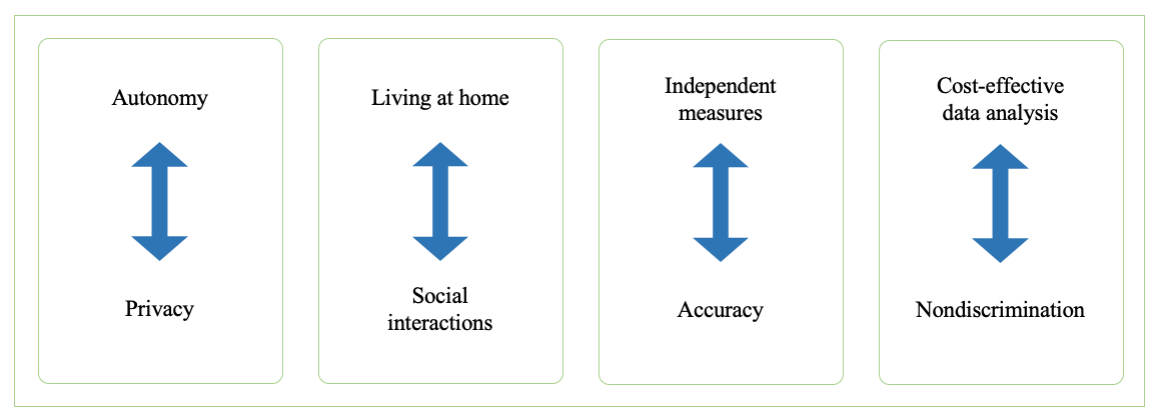
Conflicting aims and values.

Our ethical analysis showed that the rehabilitation of geriatric patients can generally be improved using smart sensors. However, we found conflicting values and aims that doctors and patients must consider when using smart sensors for rehabilitation. The use of smart sensors involves 4 pairs of conflicting ethical values and aims, which patients should sufficiently understand to provide informed consent and maintain compliance with optimal use (Figure 2).

Proactive participation in diagnostics and gaining independence can increase patient autonomy. However, gains in autonomy come at the cost of privacy. Owing to the continuous monitoring of patients’ daily activities, privacy can be violated if sensors are too intrusive, and patients have no control over their data. Moreover, when patients are aware that they are being surveilled, they may refrain from doing certain things that they value.

In contrast to smart sensors in dementia care, sensors that are used in rehabilitation are not intended to be used for surveillance, but for promoting autonomy by assisting rehabilitation measures. Increased autonomy and the benefits of home monitoring conflict with protecting patients’ privacy. The treatment team receives sensitive information using sensor technology in everyday life. The use of wearable technologies carries the risk of increasing the intended or unintended disclosure of sensitive health information [52]. This information is not consciously shared by patients with a specific health professional but is automatically collected by a technical instrument. It might not always be clear to patients who has access to this information and what the data reveal. To guarantee confidentiality of the information collected by sensors, authorized recipients must be specifically defined.

It is important that the patient be educated about the extent of the invasion of their privacy. Therefore, medical professionals must understand what conclusions can be drawn from the data in addition to the daily activity patterns. It is crucial to keep in mind that future developments could allow further data analysis and thus reveal unforeseen information, which could extend the invasion of patient privacy. Smart sensors are an attractive target for cyberattacks, because they collect valuable data and are often used in unprotected private settings. To protect the privacy of patients, it is important that service providers protect the data from unauthorized access and misuse. Regular secure backups, anonymization of the data, and limiting remotely accessible data can reduce the risk of data theft. Patients must be adequately informed and educated about this risk, ways to reduce it, and how they can avoid being monitored when privacy is desired [42].

Studies have shown that most patients do not feel that their privacy is violated by the use of smart sensors and are willing to give up some of their privacy for increased autonomy [28,41,53]. Depending on the amount of autonomy gained and the degree of invasion of privacy, there is a different level of willingness to use this technology. Older people who have an increased risk of falls or who would benefit from rehabilitative measures could consent to the invasion of their privacy by motion sensors in exchange for increased safety and autonomy [37,47]. In contrast, less vulnerable patients may have fewer reasons to allow wider intrusion in their personal life. Overall, patients need to weigh the autonomy gained with the use of smart sensors against eventual losses of autonomy by feeling compelled to adapt their behavior when monitored.

Independent measurements are the principal reason for using smart sensors at home and for monitoring daily activities; however, if patients are not sufficiently trained in the use of the sensors, it can lead to decreased accuracy of the data. Independent measures can increase patient autonomy and provide the opportunity to monitor daily activity patterns; however, they come at the cost of a decreased number of social interactions with medical professionals and reduced accuracy. Independent measurements provide the opportunity to live longer at home and generate objective data that represent daily activity patterns, but they could reduce the number of social interactions with medical professionals.

It is important that the sensor technology and underlying algorithm be supportive and not replace the diagnostic process. Before deciding for or against an intervention, the treatment team should have direct contact with the patient [54]. Sensors can support medical professionals’ subjective assessments of care needs using objective data [43]. The treatment team should critically question and contextualize the algorithmic output at any time [17]. As smart sensors reduce the number of social contacts with medical professionals, it is important to keep in mind that solitude is one of the largest welfare and mental health issues among older adults [55]. Although medical treatment may be the only social activity of a significant number of older adults, it should be noted that such interactions do not solve the problem of solitude. Better alternatives outside the therapeutic context should be offered for public mental health.

To improve the accuracy of the sensor technology, developers need to work on the accuracy of smart sensors if they are used for monitoring patients at risk. Medical professionals need to be aware that some measures that require high precision may need to be carried out under their direct supervision and that there are limits on what can be accurately measured outside clinical settings. Training should be given on the correct application of the sensors to empower the patient to increase the accuracy of the measurements.

To justify the use of public health resources, it is necessary to prove the increased effectiveness of sensor technology compared with conventional methods. A cost-effectiveness calculation of the use of smart sensors needs to fully recognize the multiple advantages of increased mobility for older adults’ well-being. In view of the long-term health benefits of increased mobility, access to smart sensors for rehabilitation should be independent of the patient’s socioeconomic status. To guarantee fair distribution, sensor technology should be prescribed by a physician and covered by health insurance. To ensure patient participation in areas with limited access, the technology should be designed such that it can be used independently or at least with the easy assistance of family members. Specialists can be contacted during anomalies [34]. We conclude that smart sensors can provide high-quality, low-cost measurement tools for many patients. However, because algorithms are seldom developed and tested for diverse populations, minorities may be at a disadvantage.

With regard to the principle of social justice, the provision of modern health care appliances for patients, such as smart sensors, requires that they are able to efficiently use them in their daily life. Smart sensors can enhance access to health care for underserved populations. However, here, as in the case of other digital instruments in health care, the opportunities provided by smart sensors are subjected to adequate use and can result in significant inequalities with respect to who can use and benefit from them [56]. The foremost is the ability to understand and use digital technologies, *digital literacy*. This ability is heterogenic and conditioned by several components; for example, skills, resources, and motivations. It has been observed that the level of literacy in the use of digital technologies is associated with social attributes of patients, such as age, level of education, health literacy in general, language barriers, immigration status, and urban or rural residence [57]. Older adult users face additional barriers when using digital technologies [58]. Extensive training and education are required regarding the use of smart sensors. Deficits in trust in digital health instruments, lack of previous experience with similar appliances, low levels of education, or language barriers can significantly impede this process.

Smart sensors have been used in geriatric rehabilitation. It has been shown that sensors can support the rehabilitation process by providing objective monitoring of a patient’s activity level [6,59]. Thus, medical professionals can define and examine rehabilitation targets along with patients to track the process. By using sensors, it is possible to compare the individual progress of a patient with the expected average progress of other patients with similar comorbidities. Activity levels can be tracked outside the therapy session. The data could be used to justify the extension of rehabilitation measures to insurance companies [6,59]. The current implementations have already addressed some of the ethical challenges mentioned in this paper, but they were used in a hospital setting. Patients were always able to communicate problems or discomfort with the sensors to medical professionals. To decrease the feeling of surveillance, the sensors were located on the lower back of the patients [6].

### Recommendations

Our principal recommendation is to consider multiple factors affecting digital literacy in the process of patient education to facilitate the effective use of smart sensors. Second, patients should not have to decide between autonomy and privacy. Developers should aim at providing solutions that promote patient autonomy while also ensuring privacy by collecting minimal amounts of data necessary to operate effectively. The standard for the ethical implementation of smart sensors should follow four prerequisites: (1) smart sensors can be activated and deactivated by the patient, (2) smart sensors are not visible to the public, (3) smart sensors only collect activity data over which a patient has control, and (4) they collect the minimal amount of data needed to allow an accurate diagnosis. In some cases, we may observe that patients refuse to sacrifice their privacy for increased autonomy. In such cases, it must be evaluated together with patients whether and to what extent this intrusion into privacy needs to be tolerated, how it can be minimized, and how great the actual benefit of sensors is for the patient in comparison with alternative treatment options.

Further recommendations for developers, patient education, health professionals, and health authorities are summarized in Textbox 1.

**Recommendations for developers, medical professionals, and health authorities.**

**Developers**

- Authorized recipients that have access to specific data must be defined.
- Data need to be protected from unauthorized access and misuse.
- Smart sensors should be activated and deactivated by the patient.
- Smart sensors should not be visible to the public.
- Smart sensors should only collect activity data over which a patient has control.
- Minimal amount of data needed to allow an accurate diagnosis should be collected.
- In order to ensure patient participation in areas with limited access, the technology should be designed so that it can be used independently, or at least easily, with the assistance of family members.
- Contact with specialists in the event of anomalies should be facilitated.

**Patient education**

- Education of the patient about the extent of invasion of privacy and the conclusions that can be drawn from the data must be done.
- Training should be given on the correct application of the sensors to empower the patient to increase the accuracy of measurements.

**Medical professionals**

- Smart sensors should augment and not replace the diagnostic process. The treatment team should have direct contact to the patient.
- Algorithmic outputs should be contextualized and questioned critically.
- Medical professionals should be aware of the limits and accuracy of smart sensors.

**Health authorities**

- It is necessary to prove an increased effectiveness of sensor technology compared with conventional methods to justify the use of public health resources. A cost-effectiveness calculation of the use of smart sensors needs to fully recognize the multiple advantages that increased mobility has for older adults’ well-being.
- To guarantee a fair distribution, sensor technology should be prescribed by a physician and covered by health insurance.

### Limitations and Comparison With Prior Work

There are already several articles that analyzed the ethical challenges of smart wearable sensors, but no article focused on smart sensors for geriatric rehabilitation [18,31]. Much of the current literature primarily discusses the ethical challenges of intelligent assistive technologies for monitoring geriatric patients, particularly in dementia care [18,60]. There are also articles that discuss issues with smart sensors used for activity and mobility monitoring. These articles focus on healthy or younger participants and rarely discuss the issues of smart sensors used by geriatric patients in rehabilitation [61,62]. Some articles discuss the use of other technologies, such as telemedicine or apps for self-management and tracking in rehabilitation [63,64]. However, these articles do not analyze the specific ethical issues associated with tools that are based on machine learning algorithms.

A limitation of this study is that it did not examine the subjective perceptions of the main stakeholders. Empirical ethical studies in the field of smart sensors are insufficient. Further work is needed to investigate the ethical insights of health professionals using smart sensors and to study the experiences of patients who use such sensors.

### Conclusions

Smart sensors offer an opportunity for the objective assessment of complex movement patterns and rehabilitation progress. Medical professionals must consider and address multiple conflicting ethical aims. One conflict in aims is that gains in autonomy often come at the cost of patient privacy. It is important that patients are educated on the insights that the collected data reveal and do not have to decide between autonomy and privacy. Furthermore, smart sensors should not replace but instead promote interaction with medical professionals. As smart sensors are complex and novel tools, medical professionals and patients should be educated on their correct applications and their limitations. Sensors should be covered by insurance to guarantee equal access to health care.
